# Associations of Preoperative Body Composition Parameters and Deltoid Cross-Sectional Area With Shoulder Function Before and After Reverse Total Shoulder Arthroplasty

**DOI:** 10.7759/cureus.117287

**Published:** 2026-10-01

**Authors:** Yuki Takahashi, Yoshihiro Hirakawa, Yuki Hasegawa, Atsushi Igawa, Kengo Inudo, Naoya Kubota, Hiroshi Kono

**Affiliations:** 1 Rehabilitation Medicine, Ishikiriseiki Hospital, Higashi-Osaka, JPN; 2 Orthopaedic Surgery, Ishikiriseiki Hospital, Higashi-Osaka, JPN

**Keywords:** bioelectrical impedance analysis, body composition, constant-murley score, deltoid cross-sectional area, japanese rehabilitation, muscle strength, phase angle, postoperative shoulder function, reverse total shoulder arthroplasty, shoulder function

## Abstract

Background

Reverse total shoulder arthroplasty (rTSA) provides favorable clinical outcomes; however, postoperative functional recovery varies among patients. Although deltoid cross-sectional area (DA) has been linked to postoperative muscle strength, the associations between systemic body composition parameters, postoperative shoulder function, and local deltoid morphology have not been fully investigated in patients undergoing rTSA. This study aimed to investigate associations between preoperative body composition parameters, DA, and shoulder function before and one year after rTSA.

Methods

This study included 42 patients (42 shoulders) who underwent rTSA and had available preoperative and one-year postoperative assessments. The study population comprised 15 men (35.7%) and 27 women (64.3%), with a mean age of 78 ± 7 years and BMI of 24.7 ± 6.4 kg/m². The underlying diagnoses were massive rotator cuff tear in 12 patients (28.6%), cuff tear arthropathy in 12 (28.6%), and glenohumeral osteoarthritis in 18 (42.9%). Six patients (14.3%) had diabetes mellitus, and 14 (33.3%) had a history of smoking. Preoperative body composition was assessed using a bioelectrical impedance analyzer (InBody® 970, InBody Japan Inc., Tokyo, Japan), including appendicular skeletal muscle mass index (ASMI), phase angle (PhA), and extracellular water/total body water (ECW/TBW). DA at the midpoint of the total deltoid length was measured using preoperative magnetic resonance imaging (MRI; Philips, Amsterdam, the Netherlands). Shoulder function was assessed preoperatively and at one year postoperatively using the Constant-Murley score, abduction strength, visual analog scale (VAS), and range of motion. Associations of preoperative body composition parameters and DA with shoulder function were assessed using Spearman's rank correlation coefficient, with the Benjamini-Hochberg procedure applied to control the false discovery rate (FDR).

Results

Preoperative PhA was significantly positively correlated with abduction strength both preoperatively (r = 0.41, adjusted p = 0.02) and at one year postoperatively (r = 0.37, adjusted p = 0.04), whereas preoperative ECW/TBW was significantly negatively correlated with preoperative abduction strength (r = −0.37, adjusted p = 0.04). Preoperative PhA was also significantly negatively correlated with VAS at one year postoperatively (r = −0.40, adjusted p = 0.03), whereas preoperative ECW/TBW was significantly positively correlated with VAS at one year postoperatively (r = 0.40, adjusted p = 0.03). Preoperative DA was significantly positively correlated with abduction strength both preoperatively (r = 0.41, adjusted p = 0.02) and at one year postoperatively (r = 0.34, adjusted p = 0.02). Furthermore, preoperative DA was significantly positively correlated with ASMI (r = 0.64, adjusted p < 0.01) and PhA (r = 0.56, adjusted p < 0.01) and significantly negatively correlated with ECW/TBW (r = −0.35, adjusted p = 0.04).

Conclusions

Preoperative body composition parameters and deltoid morphology were associated with different aspects of shoulder function before and one year after rTSA. PhA and ECW/TBW were associated with selected measures of muscle strength and pain, whereas DA was associated with abduction strength. These findings suggest that systemic body composition and local deltoid morphology may provide complementary information regarding shoulder function in patients undergoing rTSA.

## Introduction

Reverse total shoulder arthroplasty (rTSA) has become an established treatment for various shoulder disorders, including massive rotator cuff tears, cuff tear arthropathy, and glenohumeral osteoarthritis [1,2]. Unlike conventional anatomic shoulder arthroplasty, rTSA alters shoulder biomechanics and relies on the deltoid to restore shoulder elevation in rotator cuff dysfunction; many patients report pain relief and improved shoulder function [3-5]. However, the extent of postoperative functional recovery varies among patients, and identifying factors associated with postoperative outcomes remains an important clinical issue.

In rTSA, the deltoid serves as the primary muscle for restoring shoulder elevation; therefore, postoperative shoulder function is considered to be highly dependent on deltoid function [3]. Deltoid cross-sectional area (DA) has been used as an imaging parameter to quantitatively assess deltoid morphology and estimate muscle mass, as it has been shown to correlate with deltoid muscle volume [6]. Furthermore, both preoperative and postoperative DA have been reported to be significantly correlated with postoperative muscle strength in patients undergoing rTSA [7], and a recent systematic review and meta-analysis also demonstrated an association between radiologically assessed deltoid parameters and postoperative strength [8].

In recent years, body composition parameters have gained attention as indicators of physical function [9,10]. Bioelectrical impedance analysis (BIA) is a noninvasive and convenient method for assessing body composition and enables the measurement of parameters such as appendicular skeletal muscle mass index (ASMI), phase angle (PhA), and extracellular water/total body water (ECW/TBW) [9-11]. ASMI is widely used as an indicator of muscle mass, whereas PhA is considered an indicator of cell membrane function and cellular health [12,13]. ECW/TBW reflects body fluid distribution and has been reported to be associated with edema and chronic inflammation [11,14]. In the field of shoulder surgery, body composition parameters have also been associated with postoperative clinical outcomes after arthroscopic rotator cuff repair [15], suggesting that systemic factors may be associated with postoperative outcomes. Previous studies in patients undergoing rTSA have investigated local deltoid morphology as well as systemic factors such as skeletal muscle mass, sarcopenia, and grip strength [8,16]. However, studies investigating the associations between systemic body composition parameters, particularly PhA and ECW/TBW, with postoperative shoulder function and local deltoid morphology in patients undergoing rTSA remain limited. Therefore, assessing systemic body composition together with local deltoid morphology may provide complementary information for a more comprehensive evaluation of patients undergoing rTSA.

Therefore, this study aimed to investigate associations between preoperative body composition parameters, deltoid cross-sectional area (DA), and shoulder function before and one year after rTSA.

## Materials and methods

Patients

This was a retrospective observational study conducted at a single institution. This study was approved by the Ishikiriseiki Hospital Ethics Committee on June 30, 2026 (approval number: 26011). We included 47 consecutive patients (47 shoulders) who underwent rTSA at our institution between January 2024 and July 2025. The inclusion criteria were preoperative body composition assessment and MRI examination, as well as the availability of shoulder function assessments preoperatively and at one year postoperatively. Patients who underwent rTSA for fractures were excluded; five patients (five shoulders) met this exclusion criterion. Consequently, 42 patients (42 shoulders) were included in the final analysis. The study population comprised 15 men (35.7%) and 27 women (64.3%), with a mean age of 78 ± 7 years, mean height of 152.4 ± 9.2 cm, mean body weight of 58.0 ± 15.9 kg, and mean body mass index (BMI) of 24.7 ± 6.4 kg/m². The underlying diagnoses were massive rotator cuff tear in 12 patients (28.6%), cuff tear arthropathy in 12 (28.6%), and glenohumeral osteoarthritis in 18 (42.9%). Regarding comorbidities, diabetes mellitus was present in six patients (14.3%), thyroid disease in one (2.4%), renal disease in three (7.1%), cardiac disease in three (7.1%), and rheumatoid arthritis in three (7.1%); no patients had neuromuscular disease. Fourteen patients (33.3%) had a history of smoking. All patients underwent rTSA using a standard deltopectoral approach. The implants used were the EQUINOXE® Reverse Shoulder System (Exactech, Inc., Gainesville, FL) in 28 patients (66.7%) and the Tornier Perform® Humeral Stem (Tornier, Inc., Bloomington, MN) in 14 patients (33.3%). All patients followed the same postoperative rehabilitation protocol. A sling was used for one week postoperatively, and passive range-of-motion exercises were initiated on the day after surgery. Physical therapist-supervised rehabilitation was provided daily during the approximately 10-day hospitalization and once weekly on an outpatient basis after discharge. Thereafter, range-of-motion exercises progressed from active-assisted to active exercises, and muscle strengthening exercises were initiated at three months postoperatively.

Body composition assessment

Body composition was assessed once preoperatively using a bioelectrical impedance analyzer (InBody® 970, InBody Japan Inc., Tokyo, Japan). The body composition parameters evaluated were ASMI, PhA, and ECW/TBW. ASMI was calculated by dividing appendicular skeletal muscle mass by height squared (kg/m²). Whole-body values were used for PhA and ECW/TBW.

Deltoid cross-sectional area assessment

DA was measured using preoperative MRI. MRI was performed using a 3.0-T Ingenia system (Philips, Amsterdam, the Netherlands), and transverse T1-weighted images were used for the assessment (repetition time, 400-700 ms; echo time, minimum; slice thickness, 2.5 mm; interslice gap, 0.25 mm; field of view, 140 × 140 mm). The measurement level for DA was determined using an anteroposterior plain radiograph. Based on a previously described method [7], deltoid length was defined as the distance between the lower edge of the acromion and the midpoint of the deltoid tuberosity, and the midpoint of this length was defined as the center of the deltoid (Figure 1, Panel a). The transverse T1-weighted MRI slice corresponding to the level of the center of the deltoid was selected, and the entire deltoid muscle was manually traced using ImageJ version 1.54g (National Institutes of Health, Bethesda, MD). The area within the traced region was calculated as the DA (mm²) (Figure 1, Panel b). DA was measured by one examiner. To assess intra-rater reliability, the same examiner repeated the measurements in all 42 patients at a one-month interval, and the intraclass correlation coefficient (ICC) was calculated. The ICC (1,1) was 0.97.

**Figure 1:**
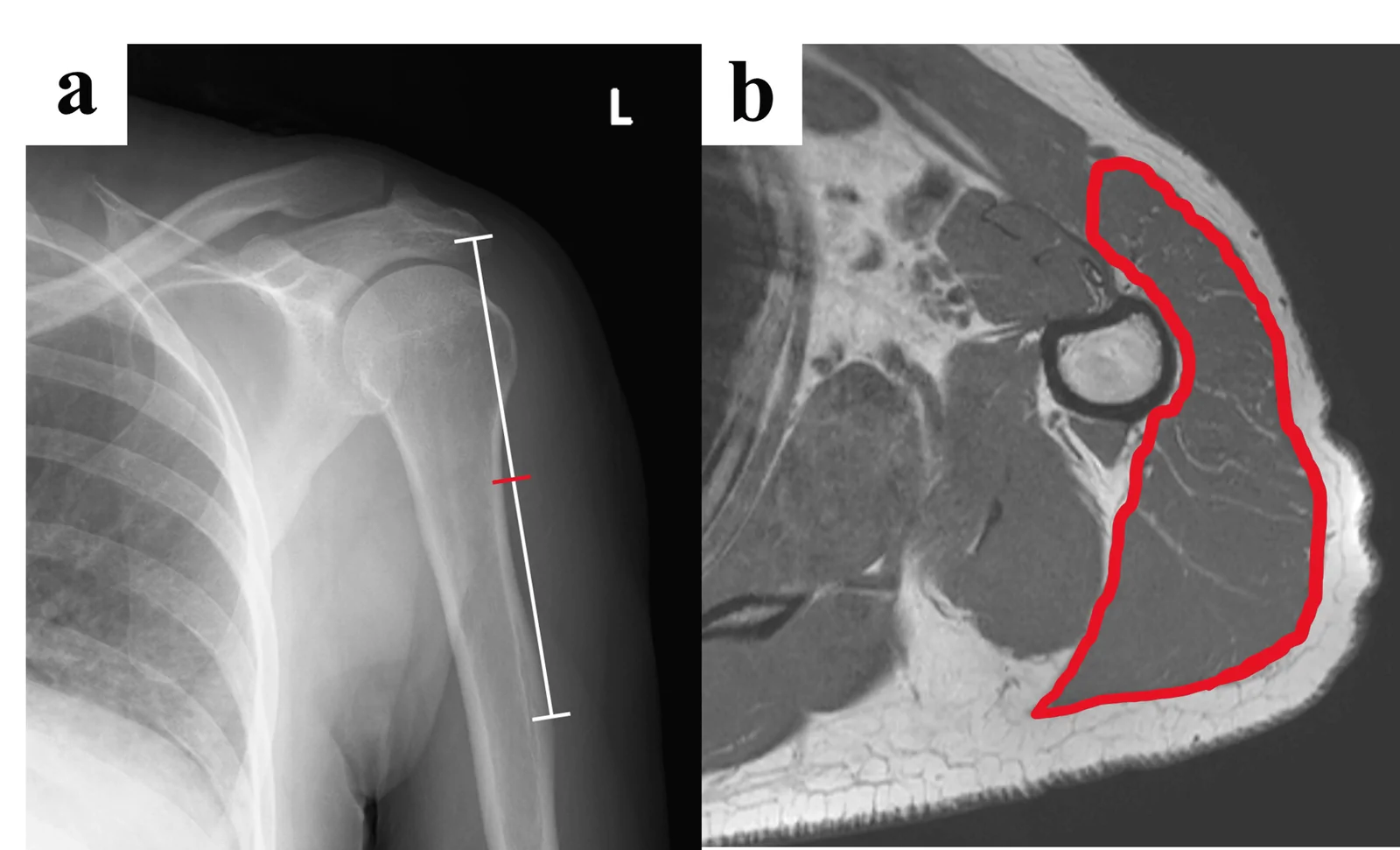
Measurement of preoperative deltoid cross-sectional area (DA) (a) Deltoid length was defined as the distance between the lower edge of the acromion and the midpoint of the deltoid tuberosity on an anteroposterior plain radiograph, and the midpoint of this length was defined as the center of the deltoid. The white line indicates the deltoid length, and the red line indicates its midpoint. (b) The entire deltoid muscle was manually traced on the transverse T1-weighted MRI slice corresponding to the level of the center of the deltoid, and the area within the traced region was calculated as the DA. Source: Original images obtained at the author's institution.

Shoulder function assessment

Shoulder function was assessed preoperatively and at one year postoperatively. The assessment included the Constant-Murley score [17], active shoulder flexion, abduction, external rotation at the side, internal rotation, abduction strength, and pain during movement assessed using the visual analog scale (VAS). Active flexion, abduction, and external rotation at the side were measured using a goniometer according to the range-of-motion measurement methods established by the Japanese Orthopaedic Association [18]. Internal rotation was assessed according to the Constant-Murley score based on the highest level reached by the thumb and was scored as follows: lateral thigh, 0 points; buttock, 2 points; lumbosacral junction, 4 points; L3, 6 points; T12, 8 points; and T7, 10 points. Abduction strength was measured with the patient standing and the affected arm positioned with the elbow extended, forearm pronated, and shoulder abducted to 90° using a handheld dynamometer (μTas F-1, ANIMA Corp., Tokyo, Japan). Two measurements were performed at maximal effort, and the higher value (lbf) was used for analysis.

Statistical analysis

The normality of continuous variables was assessed using the Shapiro-Wilk test. As some variables were not normally distributed, nonparametric statistical methods were used. Preoperative and one-year postoperative shoulder function were compared using the Wilcoxon signed-rank test. The associations between preoperative body composition parameters (ASMI, PhA, and ECW/TBW) and shoulder function preoperatively and at one year postoperatively, between preoperative DA and shoulder function preoperatively and at one year postoperatively, and between preoperative DA and preoperative body composition parameters were assessed using Spearman's rank correlation coefficient. These correlation analyses were conducted as exploratory analyses. To account for multiple comparisons, the Benjamini-Hochberg procedure was applied separately to each set of correlation analyses to control the false discovery rate (FDR), and adjusted p-values are reported. An adjusted p-value < 0.05 was considered statistically significant. All statistical analyses were performed using R version 3.6.1 (R Foundation for Statistical Computing, Vienna, Austria).

## Results

Preoperative body composition parameters, DA, and shoulder function preoperatively and at one year postoperatively are shown in Table 1. The Constant-Murley score, flexion, abduction, external rotation, abduction strength, and VAS significantly improved at one year postoperatively compared with preoperative values, whereas no significant difference was observed in internal rotation.

**Table 1:** Preoperative body composition, deltoid cross-sectional area, and shoulder function before and one year after rTSA Values are presented as mean ± standard deviation (SD). Bold values indicate statistical significance (p < 0.05). ASMI: Appendicular skeletal muscle mass index; ECW/TBW: Extracellular water/total body water; PhA: Phase angle; DA: Deltoid cross-sectional area; VAS: Visual analog scale; rTSA: Reverse total shoulder arthroplasty; —: Not assessed.

Parameter	Preoperative	One-year postoperative	p-value
ASMI (kg/m^2^)	6.3 ± 1.1	―	
ECW/TBW	0.398 ± 0.011	―	
PhA (°)	4.1 ± 0.8	―	
DA (mm^2^)	1989 ± 682	―	
Constant-Murley score	27.6 ± 15.4	62.2 ± 12.0	<0.01
Flexion (°)	79 ± 43	126 ± 20	<0.01
Abduction (°)	70 ± 39	119 ± 27	<0.01
External rotation (°)	19 ± 21	31 ± 17	<0.01
Internal rotation (points)	3.9 ± 2.1	4.6 ± 1.9	0.12
Abduction strength (lbf)	1.8 ± 2.2	5.3 ± 2.9	<0.01
VAS (mm)	68 ± 21	13 ± 18	<0.01

The correlations between preoperative body composition parameters and shoulder function preoperatively and at one year postoperatively are shown in Table 2.

**Table 2:** Correlations between preoperative body composition parameters and preoperative and one-year postoperative shoulder function P-values were adjusted using the Benjamini-Hochberg procedure to control the false discovery rate (FDR). Bold values indicate statistical significance (adjusted p < 0.05). ASMI: Appendicular skeletal muscle mass index; PhA: Phase angle; ECW/TBW: Extracellular water/total body water; VAS: Visual analog scale.

Shoulder function	ASMI		PhA		ECW/TBW	
	r	p-value	r	p-value	r	p-value
Preoperative						
Constant-Murley score	-0.05	0.78	0.27	0.14	-0.30	0.09
Flexion	-0.24	0.20	0.12	0.53	-0.21	0.25
Abduction	-0.08	0.67	0.18	0.34	-0.21	0.24
External rotation	-0.23	0.20	0.02	0.91	0.01	0.95
Internal rotation	-0.05	0.79	0.26	0.16	-0.29	0.11
Abduction strength	0.30	0.09	0.41	0.02	-0.37	0.04
VAS	0.15	0.41	-0.09	0.62	0.12	0.52
One-year postoperative						
Constant-Murley score	-0.15	0.43	0.06	0.76	-0.09	0.66
Flexion	-0.12	0.55	0.12	0.52	-0.09	0.66
Abduction	-0.16	0.41	0.26	0.18	-0.30	0.11
External rotation	-0.09	0.63	0.25	0.19	-0.25	0.19
Internal rotation	-0.15	0.45	-0.01	0.98	-0.11	0.56
Abduction strength	0.22	0.24	0.37	0.04	-0.27	0.15
VAS	0.21	0.27	-0.40	0.03	0.40	0.03

Preoperatively, PhA was significantly positively correlated with abduction strength (r = 0.41, adjusted p = 0.02; Figure 2, Panel A), whereas ECW/TBW was significantly negatively correlated with abduction strength (r = −0.37, adjusted p = 0.04; Figure 2, Panel B).

**Figure 2:**
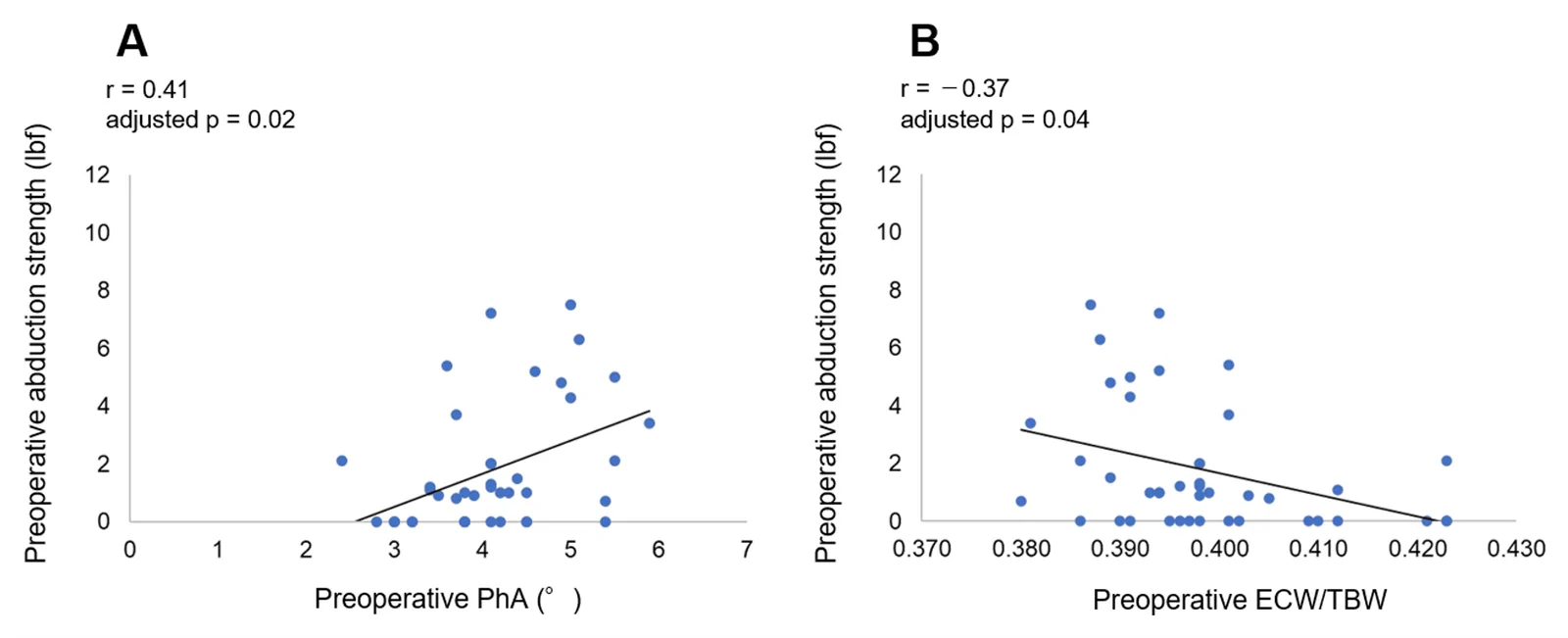
Associations between preoperative body composition parameters and preoperative abduction strength Scatter plots illustrate the relationships between variables with statistically significant correlations. Solid lines represent linear regression fits. Spearman's rank correlation coefficients (r) and adjusted p-values are shown in each panel. (A) Preoperative phase angle (PhA) and preoperative abduction strength. (B) Preoperative extracellular water/total body water ratio (ECW/TBW) and preoperative abduction strength.

At one year postoperatively, PhA was significantly positively correlated with abduction strength (r = 0.37, adjusted p = 0.04; Figure 3, Panel A) and significantly negatively correlated with VAS (r = −0.40, adjusted p = 0.03; Figure 3, Panel B). ECW/TBW was also significantly positively correlated with VAS (r = 0.40, adjusted p = 0.03; Figure 3, Panel C). No other significant correlations were observed.

**Figure 3:**
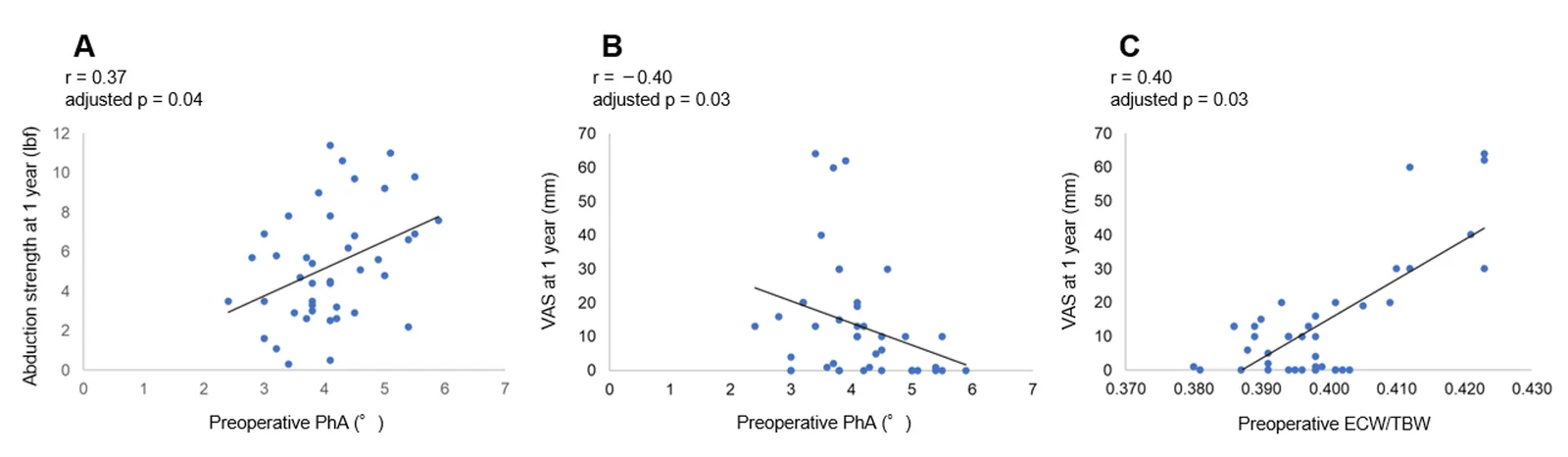
Associations between preoperative body composition parameters and abduction strength and pain at one year after rTSA Scatter plots illustrate the relationships between variables with statistically significant correlations. Solid lines represent linear regression fits. Spearman's rank correlation coefficients (r) and adjusted p-values are shown in each panel. (A) Preoperative phase angle (PhA) and abduction strength at one year after rTSA. (B) Preoperative PhA and visual analog scale (VAS) score at one year after rTSA. (C) Preoperative extracellular water/total body water ratio (ECW/TBW) and VAS score at one year after rTSA.

The correlations between preoperative DA and shoulder function preoperatively and at one year postoperatively are shown in Table 3. Preoperative DA was significantly positively correlated with abduction strength both preoperatively (r = 0.41, adjusted p = 0.02; Figure 4, Panel A) and at one year postoperatively (r = 0.34, adjusted p = 0.02; Figure 4, Panel B). No significant correlations were observed between preoperative DA and the other measures of shoulder function.

**Table 3:** Correlations between preoperative deltoid cross-sectional area and preoperative and one-year postoperative shoulder function P-values were adjusted using the Benjamini-Hochberg procedure to control the false discovery rate (FDR). Bold values indicate statistical significance (adjusted p < 0.05). VAS: Visual analog scale.

Shoulder function	r	p-value
*Preoperative*		
Constant-Murley score	0.21	0.24
Flexion	0.06	0.74
Abduction	0.14	0.44
External rotation	-0.05	0.80
Internal rotation	0.17	0.37
Abduction strength	0.41	0.02
VAS	0.05	0.80
*One-year postoperative*		
Constant-Murley score	0.06	0.75
Flexion	0.06	0.77
Abduction	0.06	0.76
External rotation	0.19	0.32
Internal rotation	0.08	0.70
Abduction strength	0.34	0.02
VAS	0.13	0.51

**Figure 4:**
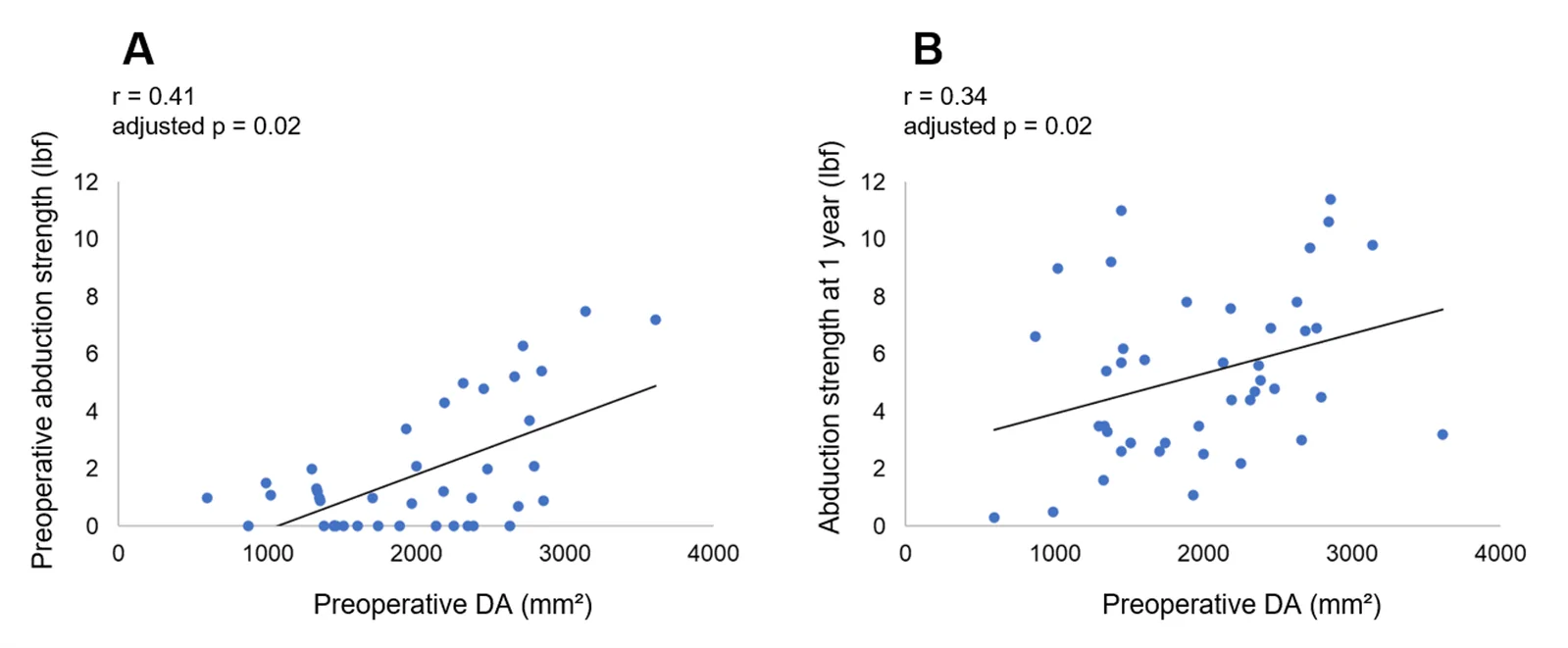
Associations between preoperative deltoid cross-sectional area and abduction strength before and one year after rTSA Scatter plots illustrate the relationships between variables with statistically significant correlations. Solid lines represent linear regression fits. Spearman's rank correlation coefficients (r) and adjusted p-values are shown in each panel. (A) Preoperative deltoid cross-sectional area (DA) and preoperative abduction strength. (B) Preoperative DA and abduction strength at one year after rTSA. rTSA: Reverse total shoulder arthroplasty; DA: Deltoid cross-sectional area.

The correlations between preoperative DA and preoperative body composition parameters are shown in Table 4. Preoperative DA was significantly positively correlated with ASMI (r = 0.64, adjusted p < 0.01; Figure 5, Panel A) and PhA (r = 0.56, adjusted p < 0.01; Figure 5, Panel B), and significantly negatively correlated with ECW/TBW (r = −0.35, adjusted p = 0.04; Figure 5, Panel C).

**Table 4:** Correlations between preoperative deltoid cross-sectional area and preoperative body composition parameters P-values were adjusted using the Benjamini-Hochberg procedure to control the false discovery rate (FDR). Bold values indicate statistical significance (adjusted p < 0.05). ASMI: Appendicular skeletal muscle mass index; PhA: Phase angle; ECW/TBW: Extracellular water/total body water.

Body composition parameter	r	p-value
ASMI	0.64	<0.01
PhA	0.56	<0.01
ECW/TBW	-0.35	0.04

**Figure 5:**
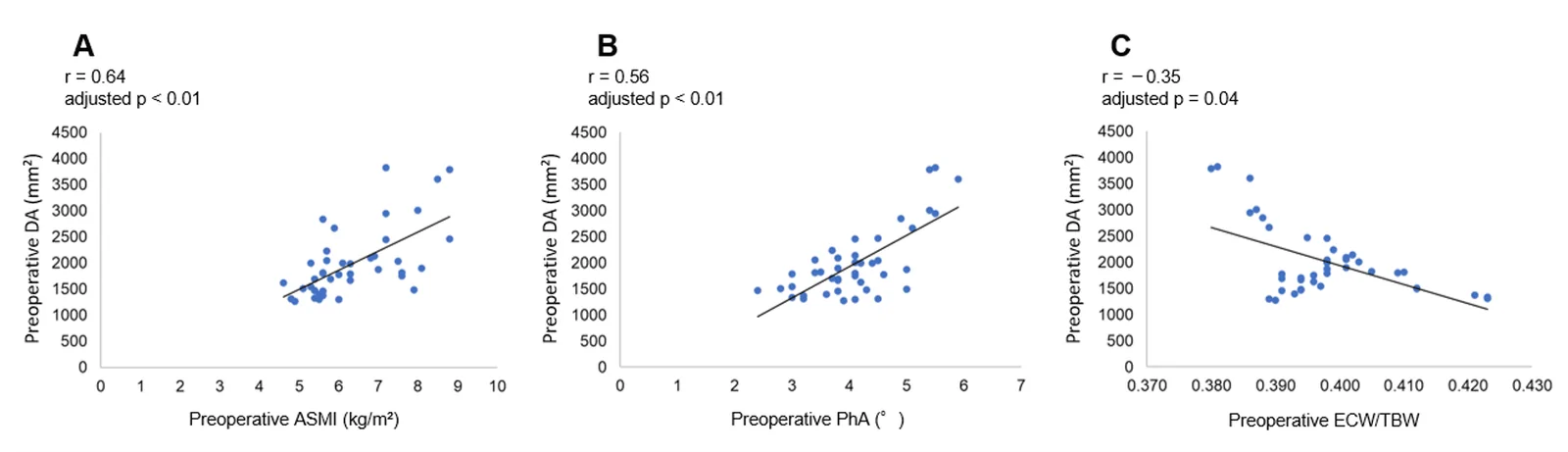
Associations between preoperative body composition parameters and deltoid cross-sectional area Scatter plots illustrate the relationships between variables with statistically significant correlations. Solid lines represent linear regression fits. Spearman's rank correlation coefficients (r) and adjusted p-values are shown in each panel. (A) Preoperative appendicular skeletal muscle mass index (ASMI) and preoperative deltoid cross-sectional area (DA). (B) Preoperative phase angle (PhA) and preoperative DA. (C) Preoperative extracellular water/total body water ratio (ECW/TBW) and preoperative DA.

## Discussion

This study investigated the associations of preoperative body composition parameters and DA with shoulder function preoperatively and at one year postoperatively in patients undergoing rTSA. Preoperative PhA and ECW/TBW were associated with preoperative abduction strength, and PhA was also associated with abduction strength at one year postoperatively. In addition, PhA and ECW/TBW were associated with pain at one year postoperatively. Preoperative DA was associated with abduction strength both preoperatively and at one year postoperatively and was significantly correlated with all body composition parameters, including ASMI, PhA, and ECW/TBW.

Regarding the correlations of preoperative PhA and ECW/TBW with postoperative pain, PhA is considered an indicator of cell membrane integrity and cellular function, and low PhA has been reported to be associated with impaired cell membrane function related to inflammation and oxidative stress [19,20]. ECW/TBW reflects body fluid distribution, and a high ECW/TBW indicates a higher proportion of ECW relative to TBW [21]. These parameters may reflect systemic qualitative aspects, such as cellular status and fluid balance, that cannot be captured by muscle mass alone. Furthermore, low PhA has been reported to be associated with elevated levels of inflammatory markers, including C-reactive protein (CRP), interleukin-6 (IL-6), and tumor necrosis factor-alpha (TNF-α) [20]. Therefore, preexisting impairment of cell membrane function and alterations in fluid distribution, together with the underlying chronic inflammatory state, may have been associated with pain at one year postoperatively.

Preoperative PhA was also correlated with abduction strength both preoperatively and at one year postoperatively. PhA has been used as an indicator of muscle quality, and an association between PhA and muscle strength has been reported in older adults [12,13]. In contrast, ASMI, which reflects muscle mass, was not significantly correlated with abduction strength in the present study. These findings suggest that, when assessing abduction strength in patients undergoing rTSA, attention should be paid not only to muscle mass but also to qualitative aspects of muscle reflected by PhA. Although ECW/TBW was correlated with preoperative abduction strength, it was not correlated with abduction strength at one year postoperatively. As ECW/TBW primarily reflects fluid balance, its association with muscle strength may be limited.

Regarding the correlation of preoperative DA with abduction strength both preoperatively and at one year postoperatively, medialization and inferiorization of the center of rotation in rTSA alter the deltoid lever arm and tension, and the deltoid plays an important role in shoulder elevation [3,7]. DA, which quantitatively assesses deltoid morphology, has been reported to be associated with deltoid muscle volume [6] and has been used as an indicator reflecting the quantitative characteristics of the deltoid. Previous studies have reported an association between DA and postoperative shoulder strength in patients undergoing rTSA [7]. A recent systematic review and meta-analysis also demonstrated a moderate association between preoperative DA and postoperative muscle strength [8], consistent with the findings of the present study.

Regarding the correlations of preoperative DA with all body composition parameters, including ASMI, PhA, and ECW/TBW, patients undergoing rTSA are generally older, and age-related systemic declines in muscle mass and muscle quality may also be reflected in the local deltoid muscle. Previous studies in patients undergoing rTSA have separately reported associations between systemic skeletal muscle mass and postoperative function [16] and between imaging characteristics of the deltoid and postoperative function [7]. However, studies directly investigating the association between MRI-assessed DA and body composition parameters in patients undergoing rTSA remain limited. The present findings suggest that deltoid muscle mass may be associated not only with systemic muscle mass but also with systemic characteristics such as cellular status and fluid balance. An advantage of BIA in the present study was that it enabled the assessment of not only muscle mass but also PhA and body fluid distribution [22]. This allowed systemic body composition to be evaluated from multiple aspects, including ASMI, PhA, and ECW/TBW, alongside local deltoid morphology assessed by MRI.

Clinically, these findings suggest that systemic body composition and local deltoid morphology may provide complementary information regarding shoulder function in patients undergoing rTSA. DA was associated with abduction strength at one year postoperatively, while PhA was associated with abduction strength and pain, and ECW/TBW was associated with pain at one year postoperatively. These findings suggest that local deltoid morphology and systemic body composition may reflect different aspects of shoulder function after rTSA. Further prospective studies are needed to clarify the clinical significance of combining these assessments.

This study has several limitations. As this was a single-center retrospective study with a relatively small sample size of 42 patients and no a priori sample size calculation, the findings should be interpreted with caution. In addition, given the limited sample size, multivariable analyses were not performed to adjust for potential confounding factors, including patient characteristics, underlying diagnoses, and comorbidities. Therefore, independent associations and causal relationships could not be determined, and the correlation analyses should be interpreted as exploratory. Another limitation is that the measurement conditions for body composition assessment using BIA were not strictly standardized. Segmental upper-extremity body composition and side-to-side differences were not evaluated, which should also be considered when interpreting the findings. In addition, body fat distribution and metabolic characteristics, including visceral adiposity and sarcopenic obesity, were not evaluated and should be considered in future studies. Furthermore, DA was assessed as the cross-sectional area on a single MRI slice and therefore did not directly represent the overall muscle volume or muscle quality of the deltoid. Although intra-rater reliability was excellent, inter-rater reliability was not assessed. In addition, absolute DA was used without normalization for sex or body size, which should be considered when interpreting the findings. Future studies with larger sample sizes that account for potential confounding factors are needed to further investigate the associations of preoperative body composition and deltoid characteristics with shoulder function after rTSA.

## Conclusions

Preoperative body composition parameters and deltoid morphology were associated with different aspects of shoulder function before and one year after rTSA. PhA and ECW/TBW were associated with selected measures of muscle strength and pain, whereas DA was associated with abduction strength. These findings suggest that systemic body composition and local deltoid morphology may provide complementary information regarding shoulder function in patients undergoing rTSA.
